# Preoperative radiotherapy of soft-tissue sarcomas: surgical and radiologic parameters associated with local control and survival

**DOI:** 10.1186/s13569-018-0106-x

**Published:** 2018-10-05

**Authors:** Panagiotis Tsagozis, Otte Brosjö, Mikael Skorpil

**Affiliations:** 1 Department of Orthopaedic Surgery, Karolinska University Hospital, Solna, Sweden; 2 Department of Molecular Medicine and Surgery, Karolinska Institutet, Stockholm, Sweden; 3 Department of Neuroradiology, Karolinska University Hospital, Solna, Sweden

## Abstract

**Background:**

Preoperative radiotherapy is often used to facilitate excision of soft-tissue sarcomas. We aimed define factors that affect local tumour control and patient survival.

**Methods:**

A single institution registry study of 89 patients with non-metastatic soft-tissue sarcomas having preoperative radiotherapy between 1994 and 2014. Radiologic (presence of peritumoural oedema and volume change following radiotherapy) and histopathologic (tumour volume, grade and surgical margin) parameters were recorded. Outcomes were the events of local recurrence, amputation, metastasis and death.

**Results:**

Local recurrence rate was low (12%) and marginal excision gave equal local control to wide excision. Pelvic localization was associated with a higher risk for amputation. The absence of peritumoural oedema on MRI defined a subgroup of tumours with more favourable oncologic outcome. Reduction of tumour volume following radiotherapy was also associated with better patient survival. Both these radiologic parameters were associated with lower tumour grade. Tumour necrosis was not significant for patient survival. The local complication rate, mainly wound healing problems and infection, was high (40%), but did not lead to any amputation.

**Conclusion:**

Preoperative radiotherapy of high-risk soft-tissue sarcomas allows for good local control rate at the expense of local wound complications, which are however manageable. Marginal excision is sufficient for local control. Absence of peritumoural oedema on MRI, as well as tumour size reduction following radiotherapy are associated to superior patient survival and can be used ass early prognostic factors.

**Electronic supplementary material:**

The online version of this article (10.1186/s13569-018-0106-x) contains supplementary material, which is available to authorized users.

## Background

Treatment of soft-tissue sarcomas is mainly surgical. Radiotherapy is indicated as an adjuvant treatment in all deep-seated tumours and in superficial tumours when a wide surgical margin is not achieved [1, 2]. It is usually given post-operatively, but may be given prior to surgery in order to facilitate tumour resection, allowing for limb-sparing surgery. Furthermore, the up-front use of radiotherapy reduces the volume of irradiated tissue, and is thought to result in a better functional outcome, but on the other hand carries a higher risk for wound complications [3]. The decision to give preoperative radiotherapy is thus individualized, taking into consideration the localization and size of the tumour, its relationship to important anatomical structures, the expected radiotherapy response and size of the radiotherapy field.

There is limited amount of data regarding the outcome of surgery preceded by radiotherapy for soft-tissue sarcomas, and there is still a debate on the factors that may determine patient prognosis. Tumour necrosis may be an objective measure of the effect of preoperative radiotherapy but there is no proof of its validity as a prognostic factor [4]. The use of radiologic measures is also questionable [5–7].

We set out to investigate the outcome of patients with soft-tissue sarcomas who were treated with radiotherapy prior to surgery, and define clinical, histologic and radiologic prognostic factors associated with survival and local control of the disease in a large retrospective series.

## Patients and methods

### Description of the cohort

This is a single-institution registry study. Inclusion criteria for participation were the diagnosis of a soft-tissue sarcoma of the trunk or the extremities, the administration of radiotherapy treatment prior to surgery and the absence of metastases at diagnosis. Exclusion criteria were chemotherapy given in a neo-adjuvant setting and a follow-up of less than 2 years for living patients. The study confirmed to Institutional Review Board requirements. The prospective database of our department was reviewed and 121 consecutive patients with a diagnosis of soft-tissue sarcoma who had preoperative radiotherapy treatment between 1994 and 2014 were identified, out of 1005 patients who had surgery for a soft-tissue sarcoma in the same time period (12%). Of these, 89 did not have any preoperative chemotherapy (usually given in the context of the SSG-XX protocol) and were finally included in this study. Patient demographics and characteristics of the cohort are presented in Table 1. Median follow-up was 5 years.

**Table 1:** Patient demographics

Age	Median: 67 years
	Range: 20–95 years
Gender	51 male
	38 female
Location	60% lower extremity
	18% upper extremity
	12% trunk
	10% pelvis
Stage (Enneking)	51% stage IIB
	31% stage IIA
	10% stage IB
	8% stage IA
Local invasion	96% deep-seated (subfacial)
	4% superficial (subcutaneous)

### Diagnosis, treatment and surveillance

Diagnosis was set in a multidisciplinary team meeting with the participation of orthopaedic surgeons, musculoskeletal radiologists, pathologists and oncologists. The decision to give preoperative radiotherapy was taken in the same meeting, with an indication to facilitate surgical resection of the tumour with an adequate surgical margin, taking into consideration the size and anatomical location of the tumour and its relationship to important structures such as the neurovascular bundle, its expected radiosensitivity and the expected morbidity related to radiotherapy and surgery. These criteria remained constant throughout the study period. Standard radiology was magnetic resonance imaging (MRI) prior to radiotherapy with another examination after given radiotherapy but prior to excision of the tumour, and a complete data set with comparable sequences prior and post radiotherapy was available for 76 patients. All MRIs were reviewed by a radiologist with many years of experience in musculoskeletal tumor imaging. Tumour dimensions (maximum dimensions in 3 axes) were measured in cm and tumour response was evaluated either as a change in tumour volume, calculated by multiplication of the maximum dimension in 3 axes, or according to the RECIST criteria using the change in the maximum diameter of the tumour, where partial response was any reduction in tumour volume ≥ 30% but with measurable tumour left, progressive disease any increase ≥ 20%, and anything else was stable disease. The degree of peritumoural oedema was subjectively evaluated in 3-grade scale (absent, moderate or heavy) using STIR and/or T2-sequences. Chest X-ray or computed tomography was used for the detection of lung metastases. Fine-needle aspiration cytology was done for diagnosis.

Radiotherapy was given as external beam photon treatment. The most common mode of radiotherapy, given in 81% of the patients, was 50 Gy given in 25 sessions of 2 Gy (5 weeks of treatment). 13% of the patients had less than 50 Gy (36–46 Gy), as a rule given in an intensity modulated treatment and 7% were treated with a dose exceeding 50 Gy (52–70 Gy). Operations were performed by consultant grade surgeons. Median time between radiotherapy and surgery was 6 weeks (range 2–28).

Surgical specimens were reviewed by a dedicated musculoskeletal pathologist. The median tumour size, as measured in the excision specimen, was 11 cm. 57% of the tumours were undifferentiated pleomorfic sarcomas, 25% liposarcomas, 8% malignant peripheral nerve sheath tumours, 7% synovial sarcomas and 3% other sarcomas.

Postoperative surveillance was according to the ESMO guidelines [8], with clinical examination and chest X-ray every 3 months for the first 2 years, every 6 months up to the 5th year after surgery, and then annually for another 5 years.

### Statistical methods

Statistics were done in the SPSS software (version 20, SPSS Inc, Chicago, IL) and the STATA (version 13). Survival analyses and comparisons were done using the Kaplan–Meier method and comparisons were done using log-rank test. Hazard ratios between groups were calculated using a Cox regression analysis (proportional hazards model), where possible prognostic factors were age (dichotomized around the median), gender, tumour grade (high or low), tumour volume (dichotomized around the median), surgical margin (wide/marginal vs intralesional), tumour necrosis (0–50%: poor response, 51–90% average response, 91–99% good response and 100% complete necrosis), and radiotherapy dose (dichotomized around the median). Competitive risk analysis was done using the method of Pepe and Mori. Chi square tests (χ^2^) were used for comparisons between groups. All tests were double-sided, and a p value of ≤ 0.05 was considered significant. 95% confidence intervals are presented in brackets. The core facility of the Statistics Department of the Karolinska Institute was consulted for the analysis of the data.

## Results

### Radiologic and histologic evaluation of the effect of radiotherapy

We first analyzed the effect that radiotherapy had on tumour volume, measured on MRI prior to radiotherapy as well as after radiotherapy (prior to surgical excision). We found that the tumour volume decreased after radiotherapy in 51% of the cases, increased in 40% and remained stable in 9%. Using RECIST criteria, stable disease was noted in 67% of cases, progressive disease in 18% and partial regression in 15%. Another radiologic parameter that could be evaluated with accuracy was the presence of peritumoural oedema. We observed that prior to radiotherapy 77% of the tumours had peritumoural oedema (67% moderate and 10% heavy), whilst after radiotherapy 82% of the tumours had peritumoural oedema (58% moderate and 24% heavy). The absence of peritumoural oedema, both prior to as well as after radiotherapy, was associated with reduction of tumour volume as evaluated by MRI (p = 0.005). However, there was no association of peritumoural oedema with partial regression according to RECIST criteria (not shown). Furthermore, tumour grade (p = 0.001), but not tumour volume (p = 0.897) was inversely correlated to the degree of peritumoural oedema. Likewise, tumour grade (p = 0.016), but not tumour volume (p = 0.089) was also inversely correlated to reduction in tumour volume after given radiotherapy. There was no correlation between the degree of volume change and the time period between given radiotherapy and the last MRI (data not shown).

Next, the degree of tumour necrosis was quantified, based on microscopic findings after excision of the tumour, since we found post-radiotherapy MRI too unreliable regarding an accurate interpretation of tissue necrosis. In 27% of the specimens necrosis was poor, in 33% average, in 24% good and in 16% complete.

Additionally, we found that the change in tumour volume had no correlation to tissue necrosis (p = 0.638). The presence or absence of peritumoural oedema prior to or after radiotherapy did not significantly correlate with the degree of tissue necrosis (p = 0.365 and p = 0.098 respectively).

### Local control rate, surgical complications and limb survival

**Fig. 1:**
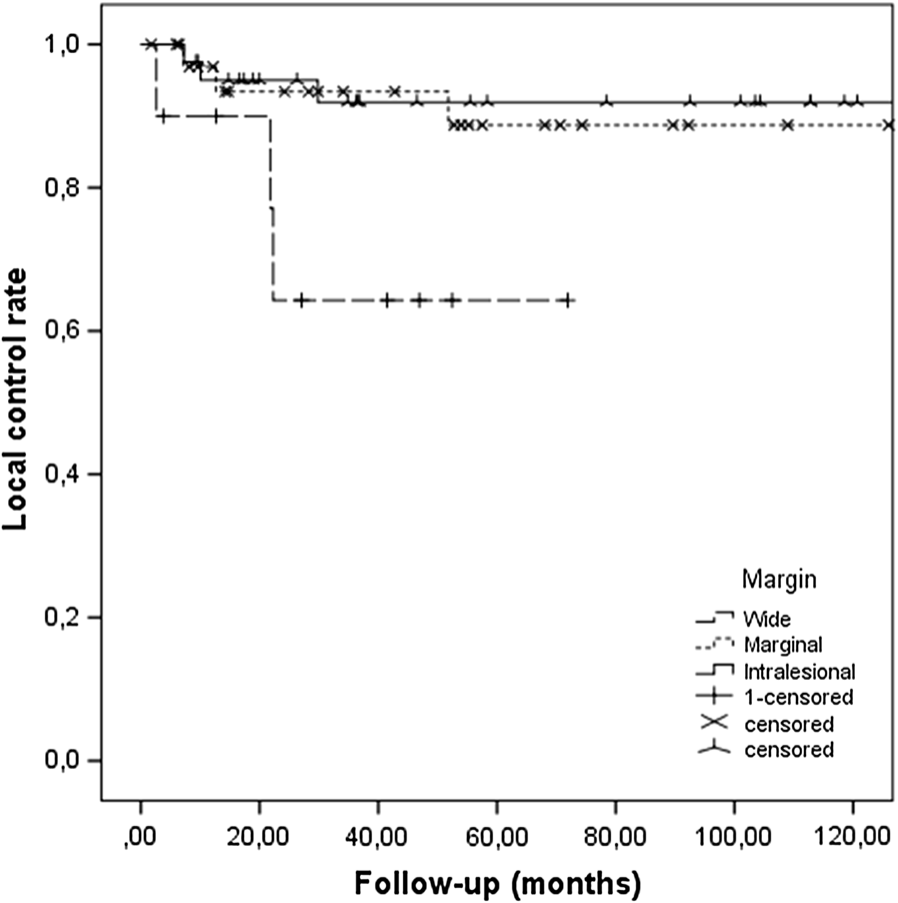
Kaplan-Meier curve of local control rate depending on the surgical margin, of patients with non-metastatic soft-tissue sarcoma of the trunk and extremities, treated with preoperative radiotherapy. Excision with clear margin provides superior local control (p = 0.014), but there is no difference between wide excisions and marginal ones

R0 surgical margins were achieved in 89% of the patients (wide in 49% and marginal in 40%, as per Enneking), whereas R1 (intralesional) margins were noted in 11%. No patients had R2 margins (intralesional with macroscopic tumour left). Local recurrence was noted in 12% of the patients. A R0 surgical margin (p = 0.014) was important for local control (Fig. 1), but there was no difference between a wide and a marginal margin. The association between clear margins and superior local control rate did not reach statistical significance during separate analysis of local recurrence with death as a competing factor (Additional file 1: Figure S1).

Complications were noted in 40% of the cases, with infections and/or wound healing problems in 36%. There were 6 grade I, 9 grade II, 19 grade III and 2 grade V complications according to the Clavien–Dindo classification. The time span between radiotherapy and surgery had no effect on local recurrence rate (p = 0.214) or the rate of wound complications. Radiotherapy dose was not associated to the rate of wound complications (p = 0.313) or local control rate (p = 0.605).

**Fig. 2:**
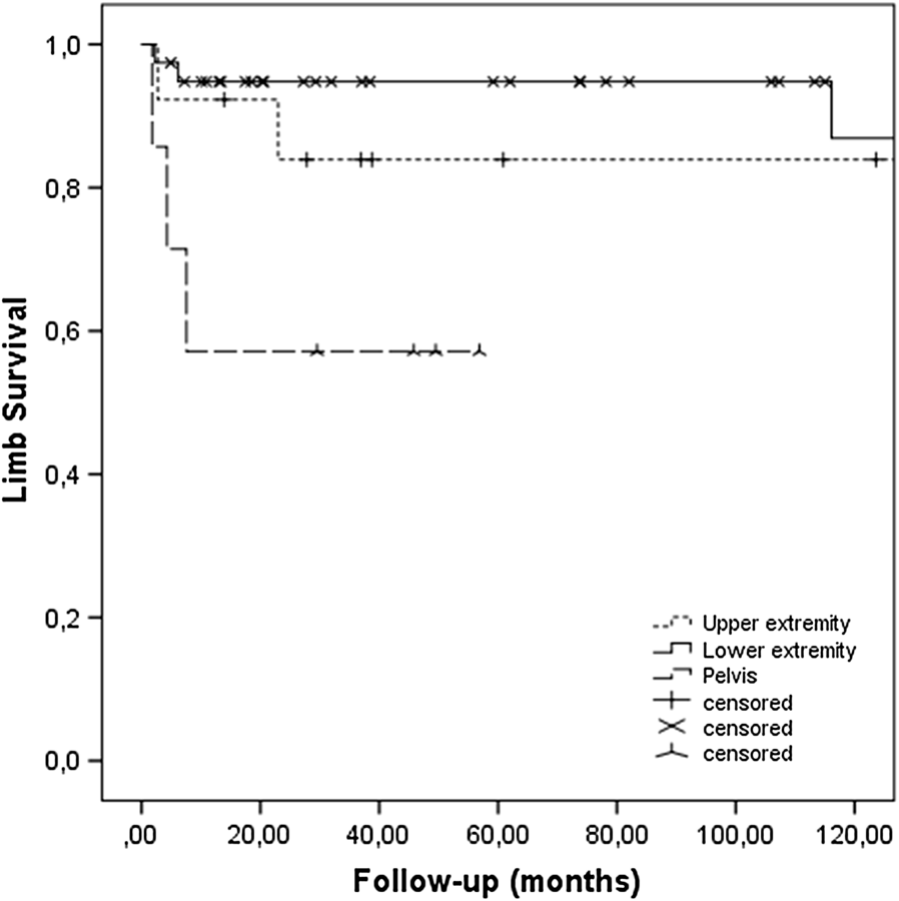
Kaplan-Meier curve of limb salvage rate regarding the upper and lower extremity, of patients with non-metastatic soft-tissue sarcoma of the extremities, treated with preoperative radiotherapy. Pelvic localization is associated with a higher risk for amputation (p = 0.029)

There were 9 amputations (in 5 patients the tumour excision was converted to amputation during their primary operation due to technical difficulty in achieving an adequate surgical margin, and 4 had secondary amputation due to local recurrence). The 5 patients who underwent a primary amputation had comparable overall survival to the rest of the patients (p = 0.099). There were no amputations due to wound healing problems and infection. Limb salvage rate was 84% at 5 years and 10 years for upper extremity tumours and 89% at 5 years and 82% at 10 years for lower extremity tumours. Pelvic location was associated with a higher risk for amputation (Fig. 2).

### Oncologic outcome and prognostic factors

Metastases were noted in 38% of the cases. The lungs were the most common localization for primary metastatic disease, as documented in 22% of the patients, whilst other atypical locations for primary metastastic disease (lymph node, skeletal and soft-tissue metastases) were relatively common in this series, as they were documented in 16% of the cases. Of the 89 patients, 31 are still alive (one with persisting tumour, the rest not having evidence of disease). Overall survival (OS) was 55% at 5 years and 44% at 10 years.

**Fig. 3:**
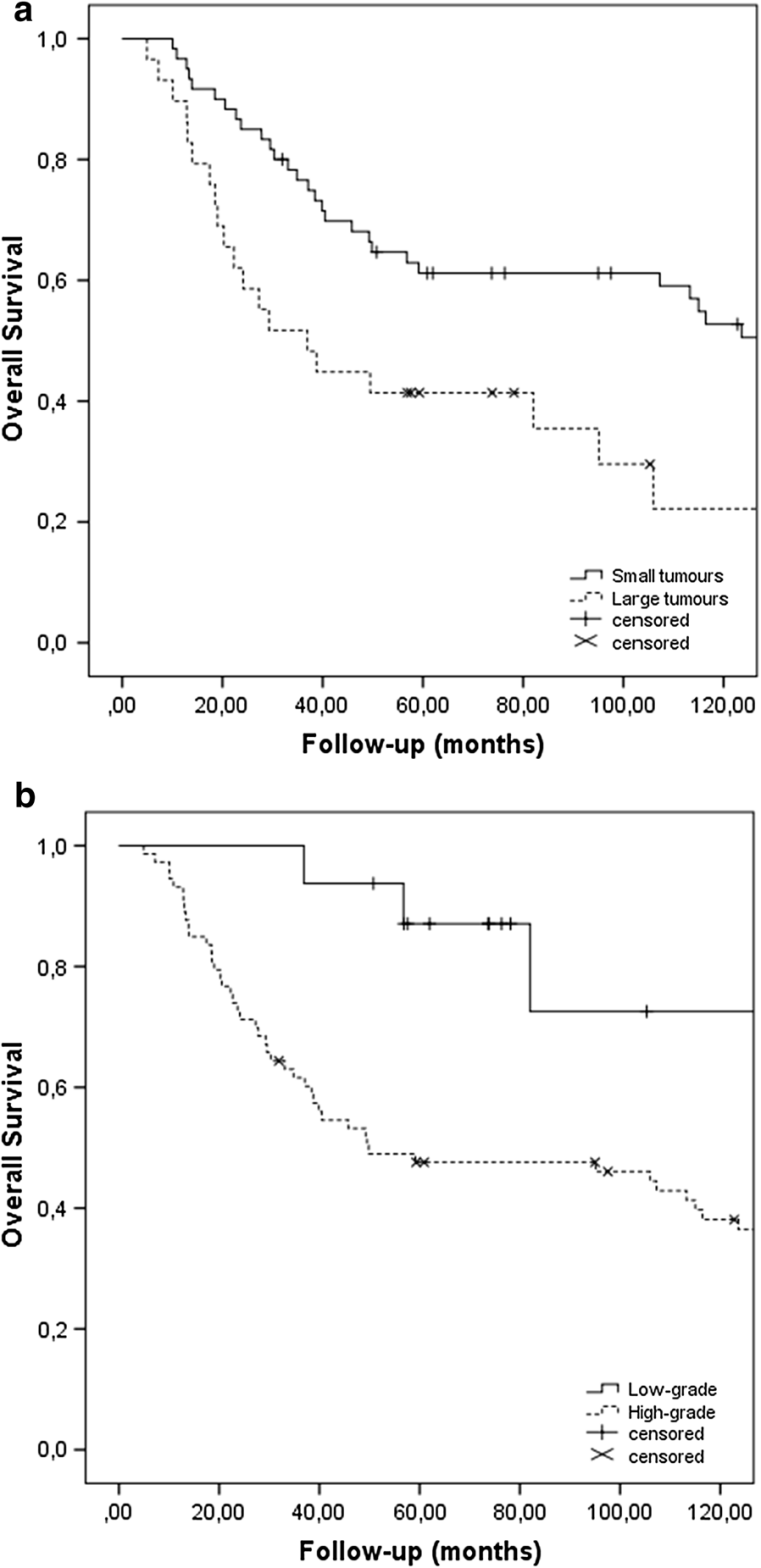
Kaplan-Meier curve of overall survival depending on tumour size (**a**) and grade (**b**), of patients with non-metastatic soft-tissue sarcoma of the extremities, treated with preoperative radiotherapy. Patients with large tumours (dichotomized around the median volume) have inferior survival to the ones having smaller tumours (p = 0.003). Higher grade is also correlated to inferior overall survival (p = 0.004)

As presented in Table 2, tumour necrosis, location and surgical margin had no effect on OS. Tumour grade, tumour size and patient age were important for OS. Tumour size (p = 0.002), grade (p = 0.028) and age (p = 0.023) retained their significance on multivariate analysis. A graphical presentation of the effect of grade and size on OS is given in Fig. 3.

**Table 2:** Overall survival

	Hazard ratio (95% CI)	p
Age	1.295–3.821	0.003
Gender	0.556–1.570	0.798
Volume	1.292–3.989	0.003
Grade	1.488–15.300	0.004
Surgical margin	0.442–2.788	0.823
Radiotherapy dose	0.543–2.303	0.762
Tumour necrosis	0.715–1.184	0.517

Effect of possible prognostic factors on the local recurrence rate as well as overall survival of patients with soft-tissue sarcomas of the trunk and the extremities that were treated with radiotherapy prior to surgery. Results gives as hazard rated with 95% confidence intervals and significance values (p)

**Fig. 4:**
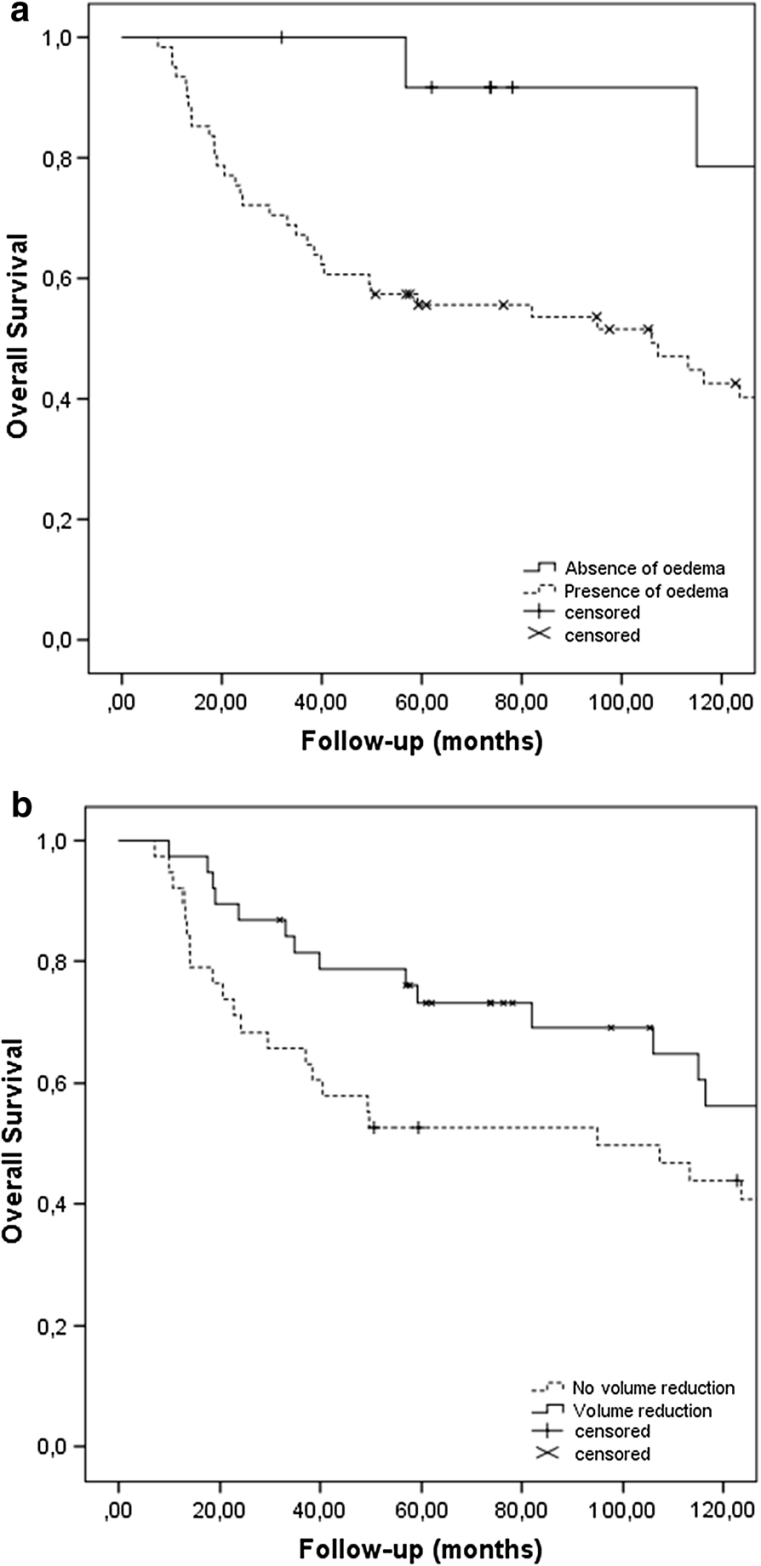
Kaplan-Meier curve of overall survival depending on the presence or absence of peri-tumoural oedema on MRI (**a**), as well as on the reduction or not of tumour size (**b**), of patients with non-metastatic soft-tissue sarcoma of the extremities, treated with preoperative radiotherapy. Absence of peri-tumoural oedema after radiotherapy (p = 0.040) and reduction of tumour volume (p = 0.015) are associated with superior overall survival

We finally tested the radiologic parameters regarding their prognostic significance (Table 2). Reduction of tumour volume,in response to radiotherapy, evaluated in absolute value, was associated with a superior oncologic outcome (Fig. 4a). In contrast, tumour response using the RECIST criteria was not prognostic for overall survival (p = 0.626). Furthermore, the absence of peritumoural oedema, best evaluated at post-radiotherapy MRI, was also a favourable prognostic factor (Fig. 4b).

## Discussion

The decision to give preoperative radiotherapy is mainly based on the intention to downsize the tumour and make it more easily resectable. Volume reduction may result in less morbidity by sparing important anatomical structures, whereas limb-sparing surgery in cases of close proximity of the tumour to the neurovascular bundle may sometimes be feasible only when preoperative radiotherapy is successful.

Our results support the notion that preoperative radiotherapy is a successful strategy in cases of high-risk tumours, such as large-volume ones and those in close proximity to the neurovascular bundle. In our cohort, average tumour size was larger than in published cohorts [1, 9], indicating that the case mix was in favour of large, high-risk tumours. R0 (wide/marginal) surgical margins were nonetheless achieved in a percentage comparable to routine sarcoma surgery [10]. Irradiated sarcomas often displayed clear anatomical margins during excision and were easy to dissect from nearby structures. This demonstrates the value of preoperative radiotherapy which is in accordance to an observed higher rate of resections with clear surgical margins in this setting [11]. The limb salvage rate was also good, although patients should be aware that in some cases the surgeon has to convert a planned limb-sparing surgery to an amputation. Pelvic localization is also an important risk factor for amputation. Importantly, there was no need to strive after wide surgical margins, since close marginal excision of the tumour gave equal local control to wide surgical excision, which is in agreement with one previous study [12]. Whereas there is no consensus regarding how radical an excision of a soft-tissue sarcoma should be [13, 14], with conflicting evidence [1, 15, 16], it appears that in the case of pre-irradiated sarcomas a close R0 surgical margin is safe.

We identified two radiologic prognostic factors that are associated with a favourable oncologic outcome, namely the absence of peritumoural oedema and the reduction of tumour volume following radiotherapy. We believe that they represent independent phenomena: The absence of oedema probably marks a more indolent biological behaviour, since there was an inverse correlation between tumour grade and the absence of oedema. Since tumour grade is, as a rule, determined with sufficient accuracy only after examination of the resection specimen, absence of peritumoural oedema can be used as an early marker to predict the oncologic outcome. Reduction of tumour volume on the other hand obviously reflects the response to treatment, although intratumoural bleeding may contribute to a stable or increasing volume and MRI sequences specific for the detection of tissue haemorrhage may be useful in this setting. Two previous studies failed to show any significance of tumour volume increase on survival [5, 6], and tumour volume reduction may be a more accurate marker. Tumour response using the RECIST criteraia was not prognostic, most probably because they are more blunt and minor volume changes are not recorded as a response. Notably, the degree of necrosis at histologic examination, another parameter which may reflect response to radiotherapy, did not correlate to the oncologic outcome, corroborating recent findings [4]. This is probably because tumour necrosis is a more complex phenomenon, which depends both on the biological aggressiveness of the neoplasm (the more aggressive and fast growing, the more necrotic) and response to treatment.

Preoperative radiotherapy was accompanied by a very high risk for local complications, often wound infections, healing problems and dehiscence, which does not depend on the time to surgery or dose. This is in line with previous publications [17–20] and should be communicated to the patient during the process of shared decision-making. The use of modern radiotherapy techniques may lower the risk of local complications [21–23]. Yet, complications were manageable and did not lead to amputations of the extremity.

We recognize the retrospective nature of this study as its main limitation. However, since our aim was not a comparison of preoperative with postoperative radiotherapy, a question which has been addressed in other studies [3, 24, 25], we consider that our study provides valuable new findings regarding preoperative radiotherapy treatment of soft-tissue sarcomas, and encourage further research in this direction so that they are validated in separate large cohorts.

## Conclusions

Preoperative radiotherapy allows for good local control of high-risk tumours and excellent limb salvage rates. This is at the expense of a considerable wound complication rate, which however does not pose a threat to limb survival. Simple marginal excision is safe and mutilating surgery to achieve a wide margin thus unnecessary. The absence of peritumoural oedema on MRI as well as volume reduction of the tumour after radiotherapy defines a subgroup of patients with favourable prognosis.

## Additional file

**Additional file 1: Figure S1.** Local relapse rate depending on the quality of surgical margins (clear or intralesional) of patients with non-metastatic soft-tissue sarcoma of the extremities, treated with preoperative radiotherapy, calculated in a competitive risk model with death as a competing factor. Clear surgical margins are not associated to local control rate in a competitive risk model (p = 0.173).
