# Supplementary material for: Preoperative radiotherapy of soft-tissue sarcomas: surgical and radiologic parameters associated with local control and survival
Source: Clin Sarcoma Res. 2018 Oct 5;8:19. doi: 10.1186/s13569-018-0106-x (PMC6172791; doi:10.1186/s13569-018-0106-x)
Supplement: Supplementary file 1 — Additional file 1: Figure S1. Local relapse rate depending on the quality of surgical margins (clear or intralesional) of patients with non-metastatic soft-tissue sarcoma of the extremities, treated with preoperative radiotherapy, calculated in a competitive risk model with death as a competing factor. Clear surgical margins are not associated to local control rate in a competitive risk model (p = 0.173). [file 13569_2018_106_MOESM1_ESM.docx]

Complementary Figure 1: Local relapse rate depending on the quality of surgical margins (clear or intralesional) of patients with non-metastatic soft-tissue sarcoma of the extremities, treated with preoperative radiotherapy, calculated in a competitive risk model with death as a competing factor. Clear surgical margins are not associated to local control rate in a competitive risk model (p=0.173).
